# Implementation of communication routines facilitating person‐centred care in long‐term residential care: A pilot study

**DOI:** 10.1111/hex.13606

**Published:** 2022-09-23

**Authors:** Emma Forsgren, Charlotta Saldert

**Affiliations:** ^1^ Institute of Health and Care Sciences, Sahlgrenska Academy University of Gothenburg Gothenburg Sweden; ^2^ University of Gothenburg Centre for Person‐Centred Care (GPCC), Sahlgrenska Academy University of Gothenburg Gothenburg Sweden; ^3^ Speech and Language Pathology Unit, Institute of Neuroscience and Physiology, Sahlgrenska Academy University of Gothenburg Gothenburg Sweden

**Keywords:** conversation, long‐term care, person‐centred care

## Abstract

**Background:**

Specific routines such as the development of personal communication plans can improve the interaction between people with communication disorders and healthcare staff. **Objective**: This pilot study explores a model for implementing communication routines including personal communication plans in long‐term residential care.

**Design, Setting and Participants:**

This multiple case study includes two residential care facilities that differ in size and the number of languages spoken. Intervention or main variables studied implementation strategies involved workshops, individual coaching and follow‐up visits. Main outcome measure implementation was assessed using goal attainment measurements, and the staff's views about facilitators and barriers to implementation were explored through structured interviews using the Theoretical Domains Framework.

**Results:**

The overall implementation success rate for the facilities was moderate, and one of the facilities showed promising results related to personal communication plans. Both facilities experienced barriers to implementation, including management problems and a lack of reminders. However, the two facilities seem to have different motivations for change.

**Discussion and Conclusions:**

Regardless of the features of the facility, successful implementation requires stable and committed leadership. Moreover, experiences with language diversity may motivate staff to implement communication routines.

**Patient or Public Contribution:**

The content of the implementation model used (i.e., choice of specific routines and implementation strategies) was coproduced by staff, managers and the researchers involved in this project. The staff and managers were responsible for the implementation of the new routines under the supervision of the researchers.

## 1 INTRODUCTION

In long‐term residential care, meaningful interaction is crucial for the well‐being of residents.^1^ However, long‐term care facilities provide limited possibilities for communication outside of care routines.^2^, ^3^, ^4^ In Sweden, only the most dependent elderly people receive residential care. Residential care residents often have hearing loss, cognitive decline due to ageing and communication disorders due to neurological disease or injury. These conditions may severely affect a person's ability both to understand and to convey their wishes and feelings. In addition, language diversity among residents as well as staff is common and this creates additional challenges for everyday interaction.^5^, ^6^

Person‐centred care (PCC) entails that the beliefs, needs and preferences of the person in need of care are respected and a partnership is established between patients and staff.^7^, ^8^, ^9^ Patients are not passive recipients of care but play an active role in planning their own care with their care providers. Studies exploring PCC in various contexts have shown that PCC has a positive effect on the cost of care, the duration of hospital stays,^10^, ^11^ patient self‐efficacy^12^ and the level of stress in healthcare staff.^13^ In PCC, the formation of personhood is seen as an activity that requires interaction with others.^14^ Therefore, a conversation partner plays a crucial role in supporting communicatively vulnerable patients.^15^, ^16^

Several researchers have assessed programmes that create routines that facilitate communication targeting healthcare staff and people with communication disorders.^17^, ^18^, ^19^, ^20^, ^21^, ^22^, ^23^ For example, Simmons‐Mackie et al.^17^ have found promising results from training in supported communication and the setting of specific goals that enhance communication access and participation for residents with aphasia. The success of communication plans has also been reported.^18^, ^19^, ^22^, ^24^, ^25^ A communication plan, which is drawn up by staff and residents together, is a summary of a resident's communicative ability and preferred communication strategies. In a study of nurses who work in a long‐term care facility for people suffering from stroke, a workshop and the development of personal communication plans resulted in nurses increasing their use of communication strategies in their interactions with residents.^19^ The staff also reported greater awareness of the importance of adapting their communication to different individuals, that is, communication became more person‐centred.

Implementation science has shown that successful and sustainable implementation in health services depends on several factors.^26^ For example, change is more likely if the staff (the intended adopters/users) want it (i.e., there is *tension for a change*).^27^ Implementation also has to *adapt to the local context*,^27^, ^28^ and some note the importance of engaging key individuals or *champions* who support the project in the organization's social networks.^29^ Moreover, the support of active and empowering managers has been found to be essential,^28^, ^30^ and giving *feedback* on the impact of the implementation increases the chance of long‐term success.^27^ However, there is no gold standard for combining implementation strategies concerning behavioural changes in staff working in residential care facilities.^31^ In addition, strategies used for implementation are sensitive to context.^31^, ^32^ Hence, before embarking on a large‐scale implementation effort, baseline facilitators and barriers to implementation need thorough exploration.^31^

Although communication plans have proven helpful, more research is needed on how to implement this kind of routine in the long‐term residential care context where residents and staff experience a variety of barriers to communication. Therefore, this pilot study explores a model for implementing communication routines, including personal communication plans for all residents, to facilitate PCC in two long‐term residential care facilities. This study has two aims: to explore the level of success in implementing personal communication plans as well as other routines supporting interaction and to present a preliminary exploration of facilitators and barriers to implementation.

## 2 METHODS

Using an exploratory and multiple‐case design, this study examines two municipal long‐term residential care facilities for older people in the same district located in western Sweden. Before the present study, both facilities had taken part in a project to implement PCC that focused on incontinence. The participating staff in this project described difficulties providing PCC for residents with communication disorders, which led them to contact the authors of the present paper. That is, the intervention was initiated by the staff from the two participating facilities and the content of the intervention was planned and coproduced with the participants in this study.

In Sweden, long‐term care is decentralized and highly subsidized as municipalities are responsible for institutional care such as residential care facilities, and approximately 95% of the cost is covered by county councils and municipalities as well as national taxes.^33^ Residents pay the remaining 5% of the cost, which includes care, rent and meals. Since the early 2000s, municipal institutional care has been seriously downsized resulting in only the most dependent older people receiving residential care (i.e., people who require more assistance and medical attention than is possible to provide in their homes). Professional care staff working in residential care facilities generally include management, registered nurses (RNs), physiotherapists and occupational therapists. However, the largest staff group is enrolled nurses (ENs) and/or nursing assistants (NAs). ENs require a secondary education in nursing (at high school) or approximately 60 weeks of postsecondary education, but NAs do not generally require any formal education. Both ENs and NAs manage daily care under the supervision of an RN. For simplicity, all the nursing staff are referred to as ENs in the following text. Typically, speech‐language pathologists are not on staff but can be consulted as part of specialist care.

### 2.1 Residential care facilities

The first facility (F1) has 20 one‐room apartments divided into three units. Two of the units offer dementia care and one offers care for those with physical disabilities. At F1, all available care staff working on the units (28 ENs) agreed to participate, and four of them became key ENs (i.e., ENs who had specific responsibility for the project; see Section 2.2 below). However, only 24 ENs provided background data (e.g., age, language background and employment) and 13 of the ENs participated in workshops included in the implementation project. All ENs who returned background data were female (20–66 years old; medium: 46 years). The ENs had 2–41 (medium: 22) years of work experience in healthcare and Swedish as their native/best language (Supporting Information: Appendix 1). One of the key ENs dropped out early in the implementation process due to sickness, and at the end of the project, none of the key ENs was still working on the unit. The unit manager (UM) at F1 went on sick leave in the middle of the implementation process and was replaced by the person responsible for planning as the assistant manager. Six of the 20 residents were more actively involved in the study (i.e., they participated in Phase 3 and provided the background information). The residents experienced a variety of barriers to communication including hearing loss, dementia and aphasia. All six participants had Swedish as their native/best language (Supporting Information: Appendix 2).

The second facility (F2) has 43 one‐room apartments divided into three units: a dementia unit, a psychogeriatric unit and a unit for those with a physical disability. There were approximately 40 ENs working on the units. At F2, 28 of 40 ENs agreed to participate, and three became key ENs. At this facility, 25 (23 females and 2 males) provided background data and 27 ENs participated in the workshop. The ENs were 22–64 years old (medium: 47 years), had 3–41 years of work experience in healthcare (medium: 19 years) and had a variety of languages as their native/first language (Supporting Information: Appendix 3). Two of the key ENs started working at night and the third key EN changed units during the implementation process. At F2, one UM was present initially, and another manager was employed just after the implementation process started. During implementation, the first manager retired, and a third manager was employed. By the end of the project, UM2 was on sick leave and her job had been taken over by a fourth temporary UM. At that time, the third UM had changed jobs. Four of 43 residents were more actively involved in the study (e.g., participated in Phase 3 and provided background information). As in F1, the residents experienced a variety of barriers to communication spanning from hearing loss to dementia. All four participants had Swedish as their native/best language (Supporting Information: Appendix 4).

### 2.2 The implementation model

**Figure 1:**
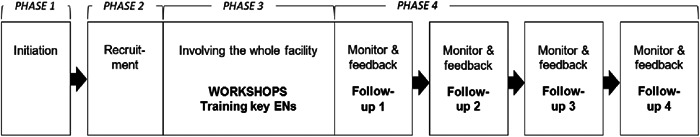
Visualization of the implementation process including the four phases

This section describes the four phases of the implementation process and the activities and strategies adopted (Figure 1).

#### 2.2.1 Phase 1—Initiation of the project

The first phase, which explored the *tension for change*, began with a meeting with the two UMs and two female speech‐language pathologists (SLPs; the authors of this paper, E. F. and C. S.). Both authors are licenced SLPs and had previous experience working with individual staff communication training in residential care facilities. At the time of the study, the first author E. F. was a PhD student and C. S. was a researcher and E.F.'s main supervisor. During this meeting, the UMs and the SLPs discussed the time plan, and unit needs concerning communication including difficulties associated with speech‐language problems were discussed. Another important issue was making the *management actively involved* and to *adapt the project* to the needs of each unit. Between 6 and 12 weeks later, short information meetings (15–30 min) were held to outline the project and introduce the ENs. The UMs then checked the degree of interest among their respective staff.

#### 2.2.2 Phase 2—Recruitment of participants and key ENs

During the second phase, the first author E. F. was present in the facilities for around 10 h per week for 2–3 weeks where they provided further information and recruited participants. During this phase, key ENs were recruited. The key ENs were responsible for the fulfilment of the unit's specific goals (see Phase 3) and served as a resource for their colleagues concerning communication matters in their units.

#### 2.2.3 Phase 3—Involving the whole facility

The third phase lasted 5 weeks and the first author E. F. was present in the facilities for approximately 15 h a week. This phase, which aimed to involve all available ENs, started with a meeting with the UM, 3–4 ENs and the authors E. F. and C. S. Preliminary goals for the whole unit were set based on the ENs' views on problems and resources. The main goal at both facilities was to draw up personal communication plans that complemented each resident's health plan. These plans were codeveloped by the ENs and residents (Supporting Information: Appendix 5). All the goals were designed to facilitate the delivery of PCC by ensuring that all residents could understand and express themselves and that all staff had general knowledge about communication disorders as well as knowledge about each resident's specific communication resources and barriers (Tables 1 and 2). In groups of 3–8, all available ENs participated in a 4 h workshop of a mixed format led by the authors E. F. and C. S. with content that had been adapted after the specific units. The workshop was based on the work described in Simmons‐Mackie et al.,^17^ Sorin‐Peters et al.^18^ and McGilton et al.^19^, ^20^ Topics covered included communication difficulties and strategies people used to address these difficulties. Core activities were watching video clips of different communication disorders and trying different strategies and resources to support communication in role‐play exercises. The unit's specific goals were further adapted during the workshops and then finalized and distributed to the UM and each of the ENs. In addition to the workshop, the key ENs had two 1‐h training sessions with the first author E. F. During these sessions, they watched and discussed video‐recorded clips of themselves interacting with a resident.

**Table 1:** Goal attainment for facility 1 at follow‐up 1, 2, 3 and 4

Unit‐specific goals	Follow‐up 1	Follow‐up 2 (4 months)	Follow‐up 3 (7 months)	Follow‐up 4 (12 months)
(1) Create personal communication plans for all 20 residents	PA 8/20 (40%)	PA 9/20 (45%)	PA 15/20 (75%)	PA 12^a^/20 (60%)
(2) Document the need for and placement of alarms	NA	PA	PA	PA
(3) Control and adjust alarm placement in apartments	NA	A	A	A
(4) Make sure the hearing loop in the common room is working and create a manual	NA	NA	PA	A
(5) Write what day it is and who is working on whiteboards in each unit every day	NA	PA	A	PA
(6) Make sure all residents who want to receive a copy of the week's menu	NA	A	A	A
(7) Create a tablet station for charging tablets	NA	NA	PA	PA
(8) Make sure tablet devices are continually updated	NA	A	A	A

Abbreviations: A, attained; NA, not attained; PA, partially attained.

^a^ At follow‐up 4, three residents who had personal communication plans had recently passed away.

**Table 2:** Goal attainment for facility 2 at follow‐up 1, 2, 3 and 4

Unit goals	Follow‐up 1	Follow‐up 2 (4 months)	Follow‐up 3 (7 months)	Follow‐up 4 (12 months)
(1) Create personal communication plans for all 43 residents	PA 0/43 (0%)	PA 1/43 (0%)	PA 7/43 (16%)	PA 6^a^/43 (14%)
(2) Make sure tablet devices are available to all staff	A	A	A	PA
(3) Demonstration of the picture material from an occupational therapist	NA	PA	PA	PA
(4) Make sure the picture material is available in each unit	NA	PA	PA	PA
(5) Create personal picture materials for at least two residents	NA	NA	PA	A
(6) Make an overview of all languages spoken in the house	NA	A	A	A
(7) Create language‐based activities for residents	NA	NA	NA	PA
(8) Make sure translation applications are available on all tablet devices	PA	PA	A	A

Abbreviations: A, attained; NA, not attained; PA, partially attained.

^a^ At follow‐up 4, one resident who had a personal communication plan had recently passed away.

#### 2.2.4 Phase 4—Monitor and feedback

In Phase 4, four follow‐up visits were conducted at both facilities—immediately after the workshops and goal setting and 4, 7 and 12 months later. The first author E. F. was on site approximately 8–15 h a week over 1–2 weeks at each follow‐up and was available if the staff requested assistance. Another important task was to *monitor* and *give feedback* on goal attainment.

### 2.3 Method for exploring success in implementation

To assess the level of success in implementing communication routines, a measure of *attained unit specific goals* was collected based on an adapted version of the Goal Attainment Scaling.^34^ This was done after the workshops and unit goal setting at follow‐up 1 and at three additional follow‐ups (4, 7 and 12 months after goal setting). The UMs in collaboration with the first author E. F. specified the steps needed to attain each goal. These steps were converted into a three‐graded scale of goal attainment. For example, for goal 5 at F1 (i.e., *Write what day it is and who is working on whiteboards in each unit every day*), the following criteria were used: if the goal was not completed in any of the three units, the goal would be considered *not attained*; if the goal was completed in one or two units, it would be seen as *partially attained* and if the goal was completed in all three units, the goal would be considered *attained*. For the main goal of creating personal communication plans, a proportion of created plans related to the number of residents was calculated.

### 2.4 Method for exploring facilitators and barriers to implementation

To explore the facilitators and barriers to implementation, formalized interviews were conducted by the first author E. F. at follow‐up 4, a year after the workshops and goal setting. At F1, one EN (who had participated in the initial planning meeting and had taken some responsibility for the unit‐specific goals) and the planner were interviewed. At F2, two key ENs (key EN1 and key EN3) were interviewed. The first author E. F. had previous experience in interviewing and had continuous contact with all the respondents during the implementation. All respondents were involved during the whole process of implementation (more or less) and agreed to an interview. The respondents have approached face‐to‐face and all accepted participation.

An interview guide was constructed using the Theoretical Domains Framework (TDF).^35^, ^36^ TDF is based on theories relevant to behaviour change in healthcare providers and it is divided into 14 categories or domains.^36^ The TDF domains were used to select 16 questions for the interview (Supporting Information: Appendix 6). The questions were not pilot tested. The interviews were conducted at the units in a private room where only the interviewer and the respondent were present. Interviews were audio recorded and lasted between 25 and 60 min. No field notes were made during and/or after the interview.

Because the interviews were guided by the TDF, a theory‐led thematic analysis was performed on the data.^37^ That is, the data were sorted into two predetermined themes—barriers to implementation and facilitators to implementation. All interviews were transcribed, and each item of information related to the theme was extracted and condensed by the first author E. F. Interrater reliability was calculated on 20% of the condensed items using blinded assessments made by a second rater (C. S.), and the two raters reached a 100% agreement on the coding of the items as either barriers or facilitators. The Consolidated Criteria for Reporting Qualitative Research (COREQ)^38^ were applied (Supporting Information: Appendix 7).

## 3 ETHICAL CONSIDERATIONS

Ethical approval for the study was obtained from the Regional Ethical Review Board of Västra Götaland in Sweden [reg no: 1016‐13]. Participants' anonymity was preserved, and they all gave their written informed consent. Participants were informed that their participation was voluntary and that they could withdraw their consent at any time. Extra care was taken in informing participating residents. They were initially approached about participating by one of the ENs working in the unit and were then asked again by the first author using picture support. The residents' significant others, family members or legal guardians were also informed about the study and gave written consent on behalf of the residents when required.

## 4 RESULTS

### 4.1 Implementation success

Analysis of *unit goal attainment* showed that F1 had attained 40% of the main goal of creating personal communication plans for all residents at follow‐up 1, 75% at follow‐up 3 and 60% at follow‐up 4. Three of the other seven unit‐specific goals were partially attained and four were completely attained at follow‐up 4 (Table 1).

At F2, the goal of creating personal communication plans was not *attained* for any of the residents directly after the workshop and goal setting (follow‐up 1), but 16% were attained at follow‐up 3 and 14% remained at follow‐up 4. Four of the seven unit‐specific goals were partially attained and three were completely attained at follow‐up 4 (Table 2).

### 4.2 Facilitators and barriers to implementation

**Figure 2:**
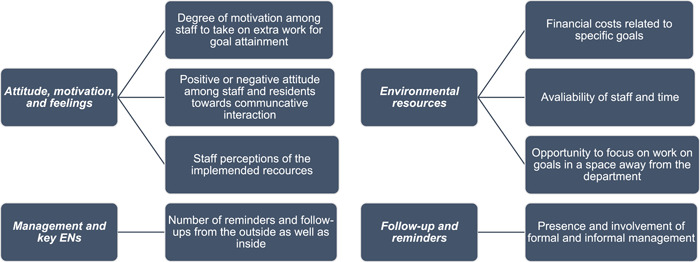
Summary of the thematic analysis of facilitators and barriers to implementation

The thematic analysis of interviews from F1 resulted in 118 items: 64 items coded as facilitators and 54 items as barriers. Analysis of interviews from F2 yielded 105 items: 75 items coded as facilitators and 30 items as barriers. All the facilitators and barriers are grouped into four subsections based on the content theme: (1) *Attitude, motivation and feelings*; (2) *Management and key ENs*; (3) *Follow‐up and reminders* and (4) *Environmental resources*. See summary in Figure 2.

#### 4.2.1 Attitude, motivation and feelings

The respondents saw the goals set as important but not demanding. However, not all staff were motivated. For example, one EN at F1 explained that not all staff had taken sufficient responsibility because some wanted to avoid additional tasks. At both facilities, staff perceiving interaction with residents as enjoyable and effective communication as crucial for accomplishing PCC were coded as facilitating. Respondents from F2 reported that it was a major problem when residents could not understand or make themselves understood due to communication disorders or language differences. It was also reported that communication problems could result in unnecessary medication administered by the staff to manage residents' anger and frustration. A barrier associated with goal 7 (*arranging activities according to language*) was that residents did not always speak the same dialect or want to socialize. The personal *communication plans* (goal 1) were described as a resource: ‘Communication plans, it's great, if it says in their files that you have a problem or what language or, it makes it easier (key EN1). Picture support materials (goal 5) were described as particularly *helpful and fun* (key EN3, F2). Pictures were also perceived as sometimes helpful when dealing with people who had a different language background (key EN1, F2).

#### 4.2.2 Management and key ENs

At both facilities, management was seen as an important factor for implementation success. However, the UMs and key ENs (who provide informal leadership) were not continuously active throughout the project. That is, most of the items relating to the management were coded as barriers.

The planner at F1 (who, by the end of the project, functioned as the assistant manager as the UM was on sick leave) talked about the importance of delegation and setting deadlines. The interviewed EN agreed: ‘It's really important that they also, you know, that they are involved throughout the process, you know, somehow they look and know which information we have received and what we know, they don't have the time, I understand of course, but it would surely be good’ (EN, F1). At F2, there were four UMs more or less involved throughout the project. The key ENs felt that the first two UMs, who initiated the project, saw it as important and assumed that it was because of these managers that the first goals were met. The key EN1 at F2 noted that the current UM's interest affects what is worked on and whether the staff continue to achieve the goals. In addition, it was also reported that two of the key ENs who were supposed to facilitate the change had begun working nights and the third had changed units.

#### 4.2.3 Environmental resources

Factors in the environment such as particular tools, staff availability and time were mentioned as both facilitating and hindering implementation. At F1, the fact that the goals were not financially costly was seen as facilitating. However, there were barriers. For example, the facility had been unable to purchase a table with wireless charging for tablets (which would have helped them meet unit‐specific goal 7). A further problem was time. At both facilities, ENs reported that taking on extra work addressing goals would negatively affect colleagues. It was also noted that it was harder to meet the unit‐specific goals that required more planning and time. At F2, the opportunity to work in peace and quiet outside of the unit was cited as important for working on goals. The key EN3 explained that there were always interruptions in the unit that made it hard to focus on extra tasks.

#### 4.2.4 Follow‐up and reminders

Follow‐up visits were conducted by the first author on four occasions. These reminders of the goals were seen as facilitating. However, barriers included the lack of additional reminders and checks.

At F1, although it was said that an outsider can ‘push to get things done’, no one on the units other than the first author followed up on goals (such as personal communication plans). The planner explained that goals can be overlooked if one is not reminded, and the EN said there should have been more reminders about the goals at unit meetings. At F2, a few reminders other than the follow‐ups done by the first author were mentioned. For example, the second UM posted notes reminding staff to complete personal communication plans together with residents.

## 5 DISCUSSION

The two facilities in this study differed in the number of residents and staff and language diversity. The model explored here was moderately successful at both facilities implementing routines that facilitate PCC. By the final follow‐up, F1 had met four of eight and F2 had met three of eight unit‐specific goals. The main goal of implementing personal communication plans was quite successful at F1. At follow‐up 3, F1 had reached a 75% goal attainment rate (whereas F2 had only reached a 16% rate). Although this had decreased to 60% at F1 at the final follow‐up, this facility showed promising results in the context of implementation science.^39^ The final follow‐up was conducted approximately 1.5 years after the implementation of the project. Implementation efforts (using appropriate design and strategies) take approximately 2–4 years to yield clear, positive results after initiation.

The two facilities showed both similarities and differences regarding the identification of facilitators and barriers to implementation. Both facilities had inconsistent management during the course of the project. Rokstad et al.^30^ propose that if the management is unable to actively help empower staff, then this task should be delegated to other formal leaders. Although the key ENs are not management or formal leaders, their status as informal leaders was one reason that they were selected for our study. Nevertheless, for various reasons, including staff turnover, these key ENs were not active throughout the project, a shortcoming that negatively impacted on‐site guidance. Regrettably, the problem of high turnover of staff and management is common in long‐term residential care.^40^ High staff turnover is associated with larger facilities and problems such as lower staffing levels and poorer quality of care. Furthermore, this high rate of turnover means heavier workloads for the remaining staff, which can lead to carers becoming burned out and ultimately the depersonalization of staff and residents.^41^ Hence, the rapid turnover of the management and staff is a serious threat to PCC. Growing workloads can also result from colleagues taking on responsibilities outside of their care routines; at both facilities, time management in relation to colleagues was identified as a barrier to goal attainment.

A difference between the two facilities was found concerning the motivation for change. At F1, although the staff thought the subject was important, they expressed no urgency about making changes^27^ as the participants did not explicitly describe communication as challenging. At F2, there was a greater interest in making changes because communication was an acknowledged problem both because of residents suffering from communication disabilities and perhaps more so because of the language diversity at the facility. Some staff also found that using picture support helped overcome language differences and this was a further motivation to work towards this goal.

We chose the two facilities for this study because they had expressed an interest in an intervention focused on communication during their participation in a previous project. The initiative to participate in the research came from the participating facilities, so the planning of the routines was mainly coproduced with the participants in the study. In an overview focused on the implementation of nursing interventions in dementia care, Karrer et al.^28^ found that implementation was more successful in organizations with a person‐centred culture and organization of care (e.g., facilities where care management is flexible and could be adapted for the persons involved). The previous project, where the two facilities in our study had been engaged, included a presentation and discussion on PCC philosophy and ethics as well as a practical application of person‐centredness in everyday care. Nonetheless, translating person‐centredness into practice is a never‐ending process and was not (as expected) completely integrated into the two facilities at the start of our project. The fact that the participants had recently been involved in another study may also have affected their motivation to work on yet another project. Nonetheless, the results of this study might be affected by our model being implemented in facilities having previous knowledge of PCC. That is, implementation work in facilities without such knowledge might result in other outcomes.

The size of each facility may have also affected the participants' sense of community and responsibility during the project. F1 was approximately half the size and had half as many ENs employed as F2. This may have encouraged a greater sense of shared responsibility for the project and may explain why F1 managed their goal attainment more effectively. At F2, however, a higher percentage of the staff group participated in the workshops than at F1, and this may also have affected their sense of responsibility. Perhaps this, together with the strong desire for change, explains why, despite many barriers, F2 still managed to attain some of the goals.

Another factor that was discussed by respondents as having an impact on implementation was the ability to actually take the time and focus on tasks connected to the facilitation of communication (e.g., producing picture materials for specific residents). In the context of residential care facilities, it has been seen that staff generally prioritize practical care tasks before aspects connected to person‐centredness (e.g., identifying residents' preferences and involving residents in making choices about their care).^42^ This type of prioritization is believed to be influenced by the staff's perceived responsibilities in their respective professions. In our study, as the majority of participants were ENs, this could potentially have had an impact on the perceived possibility to focus on aspects of communication support and facilitation.

In implementation research, there is debate about whether a single or multiple implementation strategies are preferable.^43^, ^44^ Our study used several strategies inspired by previous research^17^, ^18^, ^19^, ^22^ such as workshops, individual training and follow‐up visits. Also, a review of the implementation of psychosocial interventions in residential care concluded that multiple implementation strategies are desirable.^32^

In relation to the broader field of implementation research, our study should be seen as one of the initial steps in a larger implementation effort as it contributes to knowledge of barriers and facilitators for implementation in the context of long‐term residential care. In a larger‐scale implementation effort, an explicit theory guiding implementation should be stated and a complete evaluation of *the particular process* of implementation conducted.^31^ Research should focus on the process and *process evaluations*.^45^ The entire implementation process should be continually monitored to assess whether it is following the plan, to explore how a particular event/activity/strategy affected participants and to identify how contextual factors interplayed with implementation.^46^ Future research should, of course, also explore the success in the use of personal communication plans for various forms of barriers to communication as well as whether the use actually improves staff members' communication and residents' perceptions of staff–resident interaction.

### 5.1 Conclusions and implications

Although neither of the facilities had managed to fully attain the goals set at follow‐up, F1 showed promising results regarding the main goal of developing personal communication plans. The success of the implementation was only monitored for 1 year and this was probably too short. Despite this limited timeframe, we could identify the rapid turnover of staff as a barrier to the development of new routines. Implementation strategies that focus on adaption to the unit and involve all staff may be facilitating but reminders are also needed. In line with previous research, our result implies that leadership and facilitation are crucial for successful implementation. Leadership on all levels (e.g., managers, key ENs and external facilitators) needs to be thoroughly planned and maintained consistently during the project. Also, the roles and responsibilities of each participant and priorities for direct care and tasks focused on communication need to be grounded at all levels in the facility. Another important lesson that can be drawn from our study is that ENs may find language diversity a more significant problem and is more motivating for finding communication solutions than the presence of acquired communication disorders. This result is important to keep in mind when introducing implementation efforts focused on communication in the context of residential care facilities in the future.

## AUTHOR CONTRIBUTIONS

Both authors Emma Forsgren and Charlotta Saldert have significantly contributed to the conceptualization and design of the study. The first author Emma Forsgren conducted the data collection and analysis. The second author Charlotta Saldert was involved as a discussion partner throughout the data collection and analysis and conducted interrater reliability assessments. The first author Emma Forsgren produced the first draft, and both authors Emma Forsgren and Charlotta Saldert contributed to the final version of the manuscript.

## CONFLICT OF INTEREST

The authors declare no conflict of interest.

## Supporting information

Supporting information.

## Data Availability

The data that support the findings of this study are available from the corresponding author upon reasonable request.
