# Supplementary material for: Implementation of communication routines facilitating person‐centred care in long‐term residential care: A pilot study
Source: Health Expect. 2022 Sep 23;25(6):2982–91. doi: 10.1111/hex.13606 (PMC9700177; doi:10.1111/hex.13606)
Supplement: Supplementary file 1 — Supporting information. [file HEX-25--s001.docx]

Appendix 1. Available background data on staff in facility 1.

| **Facility 1** | | | | | | | | |
| --- | --- | --- | --- | --- | --- | --- | --- | --- |
| *Staff* | *Sex* | *Age* | *Occupation* | *Native language* | *Best*  *language* | *Years in health care* | *Years in facility* | *Employment* |
| 1 | F | 50 | EN | swe | swe | 28 | 19 | Full time |
| 2 | F | 42 | EN | swe | swe | 21 | 21 | Full time |
| 3 | F | 52 | EN | swe | swe | 34 | 14 | Part time |
| 4 | F | 42 | NA | swe | swe | 16 | <1 | Part time |
| 5 | F | 59 | NA | fin | swe/fin | 28 | 22 | Part time |
| 6 | F | 44 | EN | swe | swe | 27 | 15 | Full time |
| 7 | F | 47 | EN | nor | swe | 25 | 22 | Part time |
| 8 | F | 53 | EN | swe | swe | 25 | 22 | Full time |
| 9 | F | 51 | EN | swe | swe/eng | 30 | 14 | Part time |
| 10 | F | 22 | EN | swe | swe | 4 | 1 | Full time |
| 11 | F | 20 | EN | swe | eng | 2 | 1 | Full time |
| 12 | F | 23 | EN | ara/swe | swe | 4 | 1 | Full time |
| 13 | F | 55 | NA | swe | swe | 27 | 22 | Part time |
| 14 | F | 57 | EN | swe | swe | 39 | 19 | Part time |
| 15 | F | 50 | EN | swe | swe | 35 | 21 | Full time |
| 16 | F | 21 | EN | per/tur | swe | 3 | 2 | Full time |
| 17 | F | 41 | EN | swe | swe | 5 | 3 | Full time |
| 18 | F | 46 | EN | som | swe | 15 | 10 | Full time |
| 19 | F | 66 | NA | swe | swe |  | 22 | Part time |
| 20 | F | 55 | EN | swe | swe | 38 | 22 | Full time |
| 21KeyEN1 | F | 59 | EN | swe | swe | 5 | <1 | Full time |
| 22 Key EN2 | F | 61 | EN | swe | swe | 41 | <1 | Full time |
| 23 Key EN3 | F | 57 | EN | swe | swe | 39 | 23 | Full time |
| 24 Key EN4 | F | 42 | EN | swe | swe | 6 | 4 | Full time |

ara: Arabic; eng: English; fin: Finnish; nor: Norwegian; per: Persian; som: Somali; swe: Swedish; tur: Turkish;

Appendix 2. Available background data on residents in facility 1.

| **Facility 1** | | | | | | | | |
| --- | --- | --- | --- | --- | --- | --- | --- | --- |
| *Residents* | *Sex* | *Age* | *Disease/*  *diagnose* | *Native language* | *Best language* | *Years post diagnose* | *Years in facility* | *Hearing (hearing aid)/*  *Eyesight (glasses)* |
| 1 | F | 82 | DAT | swe | swe | - | 1 | w/o remark |
| 2 | M | 80 | DAT/  vascular damage | swe | swe | 2 | 2 | w/o remark |
| 3 | F | 83 | Dementia (unspecif.) | swe | swe | - | 1 | Severe hearing loss (hearing aid not used)/  Reading glasses |
| 4 | F | 81 | DAT | swe | swe | 1 | 3 | Reduced eyesight (glasses) |
| 5 | M | 94 | Reduced eyesight and hearing | swe | swe | - | 1 | Hearing loss (-)/ Reduced eyesight (-) |
| 6 | F | 85 | Multiple strokes | swe | swe | 10 | 10 | w/o remark |

DAT: Dementia of the Alzheimer type; swe: Swedish; -: missing data

Appendix 3. Available background data on staff in facility 2.

| **Facility 2** | | | | | | | | |
| --- | --- | --- | --- | --- | --- | --- | --- | --- |
| *Staff* | *Sex* | *Age* | *Occupation* | *Native language* | *Best language* | *Years in health care* | *Years in facility* | *Employment* |
| 1 | F | 49 | EN | spa | spa/swe | 28 | 24 | Full time |
| 2 | F | 63 | EN | tir | tir/swe | 16 | 9 | Full time |
| 3 | M | 60 | EN | per | per | 20 | <1 | Full time |
| 4 | F | 51 | NA | ara | ara/swe | 5 | <1 | Full time |
| 5 | F | 50 | EN | ara | ara/swe | 16 | 9 | Full time |
| 6 | F | 46 | EN | alb | swe | 8 | 8 | Full time |
| 7 | F | 34 | EN | amh | amh/eng | 4 | 2 | Part time |
| 8 | F | 31 | EN | hun | hun | 5 | 3 | Full time |
| 9 | M | 29 | EN | ibo | eng | 8 | 3 | Full time |
| 10 | F | 64 | EN | swe | swe | 41 | 21 | Full time |
| 11 | F | 60 | EN | swe | eng | 20 | 12 | Full time |
| 12 | F | 49 | EN | swe | swe/eng | 33 | 32 | Full time |
| 13 | F | 50 | EN | pol | swe/pol | 20 | 3 | Full time |
| 14 | F | 24 | EN | kur | swe | 5 | 2 | Full time |
| 15 | F | 41 | EN | kur | kur/ara | 12 | 10 | Full time |
| 16 | F | 42 | EN | kur | per | 21 | - | Full time |
| 17 | F | 41 | EN | vie | swe | 21 | 8 | Full time |
| 18 | F | 55 | EN | per | per/tur | 25 | 11 | Full time |
| 19 | F | 55 | EN | swe | swe | 36 | 21 | Full time |
| 20 | F | 63 | EN | chi | kin | 35 | 19 | Full time |
| 21 | F | 36 | EN | amh | swe | 17 | 8 | Full time |
| 22 | F | 56 | EN | lug | eng | 31 | 21 | Full time |
| 23 Key EN1 | F | 46 | EN | ara/per/kur | ara | 15 | 15 | Full time |
| 24 Key EN2 | F | 50 | EN | swe | swe/eng | 33 | 14 | Full time |
| 25 Key EN3 | F | 22 | EN | kur/ara | kur/swe | 3 | 2 | Full time |

alb: Albanian; amh: Amharic; ara: Arabic; chi: Chinese; eng: English; hun: Hungarian; ibo: Igbo; kur: Kurdish; lug: Luganda; per: Persian; pol: Polish; Spa: Spanish; swe: Swedish; tir: Tigrinya; vie: Vietnamese.

Appendix 4. Available background data on residents in facility 2.

| **Facility 2** | | | | | | | | |
| --- | --- | --- | --- | --- | --- | --- | --- | --- |
| *Residents* | *Sex* | *Age* | *Diagnose* | *Native language* | *Best language* | *Years post diagnose* | *Years in facility* | *Hearing (hearing aid)/*  *Eyesight (glasses)* |
| 1 | M | 86 | DAT/ multiple illnesses | swe | swe | 4 | 4 | Hearing loss (-)/  Reading glasses |
| 2 | M | 86 | Vascular dementia/ multiple illnesses | swe | swe/  eng | 1 | 1 | Reduced eyesight  (no glasses) |
| 3 | F | 81 | Mixed dementia | swe | swe | 3 | 2 | Reduced eyesight (glasses) |
| 4 | F | 84 | Multiple illnesses | swe | swe | - | <1 | Hearing loss  (no hearing aid) |

DAT: Dementia of the Alzheimer type; eng: English; swe: Swedish; -: missing data

Appendix 5. Template of a personal communication plan translated into English.

Communication plan

| Name: ……………………………………………………………………………………….  Has been filled in with the help of: …………………………………………………………  Date: ………………………………………………………………………………………… |
| --- |

Short summary:

| My communication:  Hearing/Eyesight:  When talking to me, keep in mind:  I like:  I don’t like:  My routines: |
| --- |

How would you describe your communication/communication difficulties?

…………………………………………………………………………………………………..

………………………………………………………………………………………………….

Do you have a hearing loss? If so, do you use any hearing aids?

………………………………………………………………………………………………….

Do you have a visual impairment? If so, do you wear glasses?

………………………………………………………………………………………………….

Do you have any cognitive aids, or other communication aids? Which?

……………………………………………………………………………………………………

…………………………………………………………………………………………………..

Is there anything I as a conversation partner should think about when talking to you?

…………………………………………………………………………………………………..

………………………………………………………………………………………………….

What do you like to do for activities/talk about/watch/listen to/interests?

……………………………………………………………………………………………………

…………………………………………………………………………………………………..

Do you need any special adaptations to be able to participate in activities? (*e.g. privacy, aids, time of day, rest before/after*)

…………………………………………………………………………………………………

…………………………………………………………………………………………………

What did you like to do/talk about/watch/listen to/interests earlier in life?

…………………………………………………………………………………………………

…………………………………………………………………………………………………

Is there a specific situation when communication works extra well? What makes it work so well?

…………………………………………………………………………………………………

…………………………………………………………………………………………………

What don’t you like to do for activities/talk about/watch/listen to?

…………………………………………………………………………………………………

…………………………………………………………………………………………………

Is there a specific situation when communication is extra problematic? What makes it difficult?

…………………………………………………………………………………………………

…………………………………………………………………………………………………

Do you have any daily routines or preferences that are important to know about? (*concerning e.g.* *hygiene, diet, participation*)

…………………………………………………………………………………………………

…………………………………………………………………………………………………

What does your family situation look like? Which people are important to you?

…………………………………………………………………………………………………

…………………………………………………………………………………………………

What was your previous life like? What special needs/preferences/wishes do you have in your everyday life?

…………………………………………………………………………………………………

…………………………………………………………………………………………………

Other information that is important to know when communicating with you:

…………………………………………………………………………………………………

…………………………………………………………………………………………………

Appendix 6. Theoretical Domains Framework (TDF) - Interview guide. TDF domains from Michie et al. 2005 and Cane et al. 2012. Domains only present in Cane et al. 2012 are italicised.

| *Interview questions* | *TDF domains* |
| --- | --- |
| 1. What was the aim of the project? | Knowledge  Skills |
| 2. What do you know about the goals set in the project?   - What were the goals about? Why did we set these particular goals? |  |
| 3. Did you learn anything in the project?   - Did you get any new knowledge, ideas or new strategies you can use in communication with the residents? If so, from whom did this new knowledge come from? From your colleagues? From us? |  |
| 4. How do you perceive your own role in the project and in your workplace?   - Is there anything in your own role that affected how you participated in the project? Do you have any thoughts on other people’s participation?   The manager's role? Your colleagues' roles? | Social/professional role and identity  Beliefs about capabilities  Environmental context and resources  Social influences (norms) |
| 5. What do you think influenced how the goals of the project could be fulfilled?   - Which aspects in yourself do you think influenced how the goals (individual/shared) in the project could be fulfilled? What facilitated? Which obstacles were there? - How do you think the environment and the resources or technical systems in the workplace affected the fulfilment of goals? What facilitated? Which obstacles were there? - How do you think the social context, i.e., between colleagues/ residents/management affected the fulfilment of goals? What facilitated? Which obstacles were there? |  |
| 6.* What was the reason behind you volunteering to be a key EN?   - How did you experience the individual training? Did you learn anything? | Beliefs about consequences  Motivation and goals/*Goals* |
| 7. What is your motivation to facilitate in communication with the residents? |  |
| 8. What was your motivation to fulfil the goals set?   - Would other goals have been more motivating? |  |
| 9. How did you perceive the carrying out of goals?   - Was it demanding? (to create Communication plans, etc.). | Memory, attention, and decision process |
| 10. How did you follow up the goal fulfilment in the workplace?   - Did you talk about the goals during unit meetings or at another time when I was not in place? Did anyone on the unit remind the others? |  |
| 12. Do you believe that the goals set will change anything in the unit or in communication with the residents?  13. Do you believe that the goals will be maintained in the future? Why/why not?  14. What do you think will be required in order to maintain the goals?   - Which conditions needs to be met? | Behavioural regulation  Nature of behaviour  *Intentions* |
| 15. What are your expectations for the future concerning communication?   - Do you expect positive things or negative? | *Optimism* |
| 16. What do you thing about taking on extra tasks at work?   - Will you be rewarded in some way? | *Reinforcements* |

*Question only posed to key-ENs

Appendix 7. COREQ (COnsolidated criteria for REporting Qualitative research) Checklist

| **Topic** | **Item No.** | **Guide Questions/Description** | **Reported in section:** |
| --- | --- | --- | --- |
| **Domain 1: Research team and reflexivity** | | | |
| *Personal characteristics* | | | |
| Interviewer/facilitator | 1 | Which author/s conducted the interview or focus group? | Method (The first author EF conducted all interviews) |
| Credentials | 2 | What were the researcher’s credentials? e.g., PhD, MD | Title page (The authors EF and CS are both SLPs and has the title of PhD) |
| Occupation | 3 | What was their occupation at the time of the study? | Method (EF was employed as a PhD-student and CS as a researcher/EFs main supervisor) |
| Gender | 4 | Was the researcher male or female? | Method (Both authors are female) |
| Experience and training | 5 | What experience or training did the researcher have? | Method (EF and CS had previous experience in working with individual staff communication training in residential care facilities as well as experience in interviewing). |
| *Relationship with participants* | | | |
| Relationship established | 6 | Was a relationship established prior to study commencement? | Method (Contact with unit managers was established before the study. Both facilities participated in a previous research study which lead to contact with the authors). |
| Participant knowledge of the interviewer | 7 | What did the participants know about the researcher? e.g., personal goals, reasons for doing the research | Method (EF had continuous contact with all respondents during the process of implementation). |
| Interviewer characteristics | 8 | What characteristics were reported about the inter viewer/facilitator? e.g., Bias, assumptions, reasons and interests in the research topic | Method (EF was an SLP/PhD-student who had been present at each facility for several weeks, held initial presentations, meetings, workshops and conducted data collection). |
| **Domain 2: Study design** | | | |
| *Theoretical framework* | | | |
| Methodological orientation and Theory | 9 | What methodological orientation was stated to underpin the study? e.g.,  grounded theory, discourse analysis, ethnography, phenomenology, content analysis | Method (Theory-led thematic analysis). |
| *Participant selection* |  |  |  |
| Sampling | 10 | How were participants selected? e.g., purposive, convenience, consecutive, snowball | Method (Purposive, people involved in the whole process of implementation). |
| Method of approach | 11 | How were participants approached? e.g., face-to-face, telephone, mail, email | Method (face-to-face). |
| Sample size | 12 | How many participants were in the study? | Method (Four respondents, two at each facility). |
| Non-participation | 13 | How many people refused to participate or dropped out? Reasons? | Method (None). |
| *Setting* |  |  |  |
| Setting of data collection | 14 | Where was the data collected? e.g., home, clinic, workplace | Method (The interviews were conducted at the respective units in a private room). |
| Presence of nonparticipants | 15 | Was anyone else present besides the participants and researchers? | Method (No). |
| Description of sample | 16 | What are the important characteristics of the sample? e.g., demographic data, date | Method/ Appendices 1 and 3 |
| *Data collection* |  |  |  |
| Interview guide | 17 | Were questions, prompts, guides provided by the authors? Was it pilot tested? | Method/ Appendix 6 (The questions were not pilot-tested). |
| Repeat interviews | 18 | Were repeat inter views carried out? If yes, how many? | No |
| Audio/visual recording | 19 | Did the research use audio or visual recording to collect the data? | Method (Interviews were audio recorded). |
| Field notes | 20 | Were field notes made during and/or after the interview or focus group? | Method (No). |
| Duration | 21 | What was the duration of the inter views or focus group? | Method (between 25-60 minutes). |
| Data saturation | 22 | Was data saturation discussed? | No |
| Transcripts returned | 23 | Were transcripts returned to participants for comment and/or correction? | No |
| **Domain 3: analysis and findings** | | | |
| *Data analysis* |  |  |  |
| Number of data coders | 24 | How many data coders coded the data? | Method (One coder coded all data. Inter-rater reliability was calculated on 20% of the condensed items). |
| Description of the coding tree | 25 | Did authors provide a description of the coding tree? | Method (Two pre-determined themes were used). |
| Derivation of themes | 26 | Were themes identified in advance or derived from the data? | Method (In advance). |
| Software | 27 | What software, if applicable, was used to manage the data? | No specific software used |
| Participant checking | 28 | Did participants provide feedback on the findings? | No |
| *Reporting* |  |  |  |
| Quotations presented | 29 | Were participant quotations presented to illustrate the themes/findings? Was each quotation identified? e.g., participant number | Results |
| Data and findings consistent | 30 | Was there consistency between the data presented and the findings? | Results |
| Clarity of major themes | 31 | Were major themes clearly presented in the findings? | Results (Two major themes where used and for the presentation of results four subsections were used). |
| Clarity of minor themes | 32 | Is there a description of diverse cases or discussion of minor themes? | No |

Developed from: Tong A, Sainsbury P, Craig J. Consolidated criteria for reporting qualitative research (COREQ): a 32-item checklist for interviews and focus groups. *International Journal for Quality in Health Care*. 2007. Volume 19, Number 6: pp. 349 – 357
